# Metabolic Volumetric Parameters in ^11^C-Choline PET/MR Are Superior PET Imaging Biomarkers for Primary High-Risk Prostate Cancer

**DOI:** 10.1155/2018/8945130

**Published:** 2018-11-05

**Authors:** Jing-Ren Tseng, Lan-Yan Yang, Yu-Chun Lin, Chung-Yi Liu, See-Tong Pang, Ji-Hong Hong, Tzu-Chen Yen, Li-Jen Wang

**Affiliations:** ^1^Department of Nuclear Medicine and Center for Advanced Molecular Imaging and Translation, Chang Gung Memorial Hospital at Linkou, Taoyuan, Taiwan; ^2^Department of Medical Imaging and Radiological Science, College of Medicine, Chang Gung University, Taoyuan, Taiwan; ^3^Biostatistics Unit, Clinical Trial Center, Chang Gung Memorial Hospital at Linkou, Taoyuan, Taiwan; ^4^Department of Medical Imaging and Intervention, Chang Gung Memorial Hospital at Linkou, Taoyuan, Taiwan; ^5^Division of Urology, Department of Surgery, Chang Gung Memorial Hospital at Linkou, Taoyuan, Taiwan; ^6^Department of Radiation Oncology, Chang Gung Memorial Hospital at Linkou, Taoyuan, Taiwan; ^7^Institute for Radiological Research, Chang Gung Memorial Hospital and Chang Gung University, Taoyuan, Taiwan

## Abstract

**Purpose:**

Positron emission tomography/magnetic resonance imaging (PET/MRI) can facilitate the use of noninvasive imaging biomarkers in clinical prostate cancer staging. Although multiparametric MRI is a widely used technique, the clinical value of simultaneous PET imaging remains unclear. This study aimed at investigating this issue.

**Methods:**

Between January 2015 and December 2016, 31 high-risk prostate cancer patients underwent ^11^C-choline PET/MRI for staging purposes. Clinical characteristics and imaging parameters, including the standardized uptake value (SUV) and metabolic volumetric parameters from PET imaging; apparent diffusion coefficient (ADC) values from diffusion-weighted imaging; and volume transfer rate constant (Ktrans), reflux rate constant (Kep), and initial area under curve (iAUC) in 60 seconds from dynamic contrast-enhanced (DCE) MRI were analyzed.

**Results:**

^11^C-Choline PET imaging parameters were significantly correlated with prostate-specific antigen (PSA) levels, and metabolic volumetric parameters, including metabolic tumor volume (MTV) and uptake volume product (UVP), showed significant correlations with other MRI parameters. In our cohort analysis, the PET/MRI parameters UVP/minimal ADC value (ADC_min_) and kurtosis of Kep (Kep_kur_)/ADC_min_ were significant predictors for progression-free survival (PFS) (HR = 1.01, 95% CI: 1.00–1.02, *p*=0.031 and HR = 1.09, 95% CI: 1.02–1.16, *p*=0.009, respectively) in multivariate Cox regression analysis. High UVP/ADC_min_ and Kep_kur_/ADC_min_ values were significantly associated with shorter PFS.

**Conclusions:**

Metabolic volumetric parameters such as MTV and UVP can be routinely used as PET imaging biomarkers to add prognostic value and show better correlations in combination with MR imaging parameters in high-risk prostate cancer patients undergoing ^11^C-choline PET/MRI.

## 1. Introduction

Prostate cancer (PCa) is the leading cause of cancer-related death among men in Taiwan, with its incidence increasing rapidly from 26.22 per 100,000 males in 2002 to 47.86 per 100,000 males in 2012 [1]. The conventional imaging modalities used to assess PCa include computed tomography (CT), magnetic resonance imaging (MRI), transrectal ultrasound (TRUS), and bone scintigraphy. However, simultaneous positron emission tomography (PET)/MRI has great potential to enhance clinical practice in these cases by combining functional, molecular, and anatomic information [2]. Although ^11^C-choline has been approved for patients with suspected PCa recurrence by the U.S. FDA, the combination of ^11^C-choline and MRI may also be effective for staging, especially in patients with a high Gleason score, advanced clinical stage, and elevated prostate-specific antigen (PSA) levels [3, 4].

Biomarkers derived from medical images offer several advantages, including ready availability, noninvasiveness, and serial patient monitoring [5]. The apparent diffusion coefficient (ADC) in diffusion-weighted MRI and the standardized uptake value (SUV) in PET have been used together as an imaging biomarker in several previous studies [6–8]. However, studies on the use of ^11^C-choline PET metabolic volumetric parameters in combination with the SUV and ADCs and dynamic contrast-enhanced (DCE) parameters from multiparametric prostate MRI, which may theoretically maximize the value of simultaneous PET/MRI assessments, in cases of high-risk PCa are still lacking. Herein, we sought to identify clinically significant integrated ^11^C-choline PET/MRI parameters for high-risk primary PCa by assessing the correlations of these parameters with clinical characteristics and progression-free survival (PFS).

## 2. Materials and Methods

### 2.1. Study Patients

**Figure 1:**
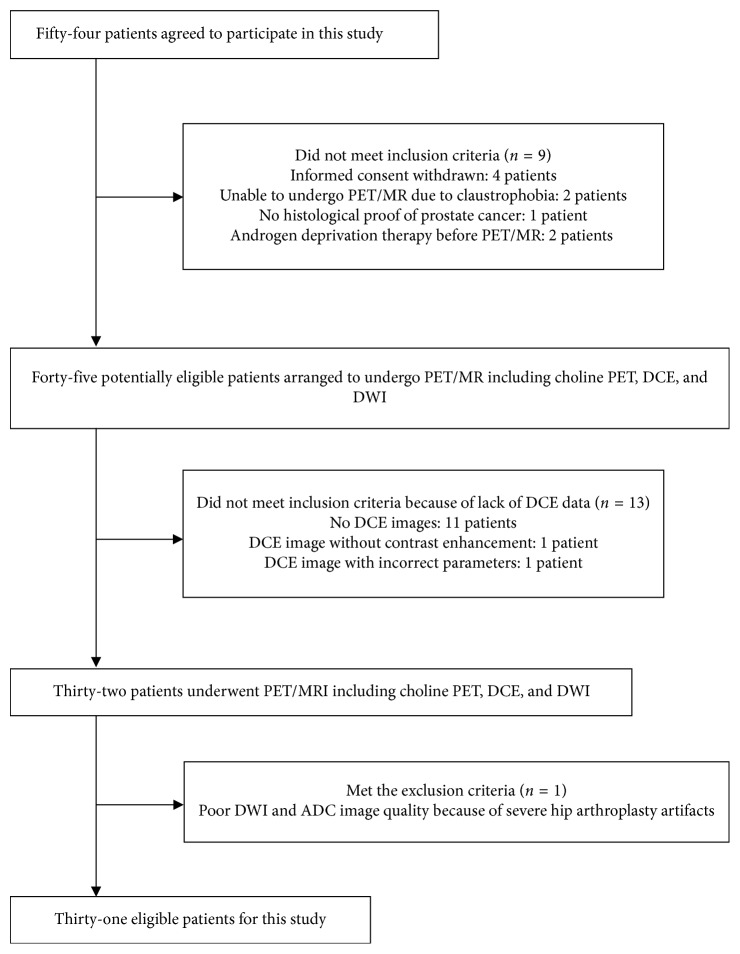
Flow diagram of the 31 patients eligible for this study.

This was a retrospective analysis of a prospective study. The study was approved by the hospital's institutional review board (approval numbers: 102-3271A and 201701793B0; ClinicalTrials.gov identifier: NCT02852122), and informed consent was obtained from all patients. Between January 2015 and December 2016, 54 consecutive patients with a clinical indication for PCa staging were scheduled to undergo an ^11^C-choline PET/MRI examination, and a total of 31 patients were eventually assessed in this retrospective analysis. The study scheme is shown in Figure 1. All 31 patients had pathologically proven high-risk prostate cancer according to the D'Amico Risk Classification [9].

We recorded primary treatments (i.e., radical prostatectomy, hormone therapy, and radiation therapy plus hormone therapy) for prostate cancer in all 31 patients, and their follow-up status after treatments. The follow-up period was until June 2018. Disease progression after primary treatments was considered if the patients showed local or nodal relapse, new metastasis, or biochemical failure (a PSA value ≥0.2 ng/ml with a secondary confirmatory PSA value >0.2 ng/ml after radical prostatectomy; PSA value >2 ng/ml above nadir after radiation therapy without or with hormone therapy; or a continuous increase in PSA values after hormone therapy) [10–12]. The follow-up durations from diagnosis to disease progression or death/last visit of all patients were recorded.

### 2.2. ^11^C-Choline PET/MRI Protocol

After fasting for at least 6 h, patients received a single intravenous bolus of 10–20 mCi (370–740 MBq) ^11^C-choline; the mean dose was 16.5 ± 3.6 mCi. Approximately 5 min after ^11^C-choline injection and bladder evacuation, whole-body PET/MRI scanning was performed using an integrated PET/MRI system (Biograph mMR; Siemens Healthcare, Erlangen, Germany). PET scans were performed from the mid-thigh to the head in five bed positions (acquisition time, 3 min per position) with the patient in a supine arms-down position. Simultaneous MRI was performed with a transverse T2-weighted half-Fourier single-shot TSE (turbo spin-echo) sequence (1,000 ms repetition time (TR)/84 ms echo time (TE), 6 mm slice thickness, 320 × 256 matrix, and 380 × 309 mm^2^ field of view (FOV)) and a coronal T1-weighted TSE sequence (500 ms TR/9.5 ms TE, 5 mm slice thickness, 1.5 mm intersection gap, 384 × 276 matrix, and 450 × 310 mm^2^ FOV), while acquiring PET data in each bed position.

**Figure 2:**
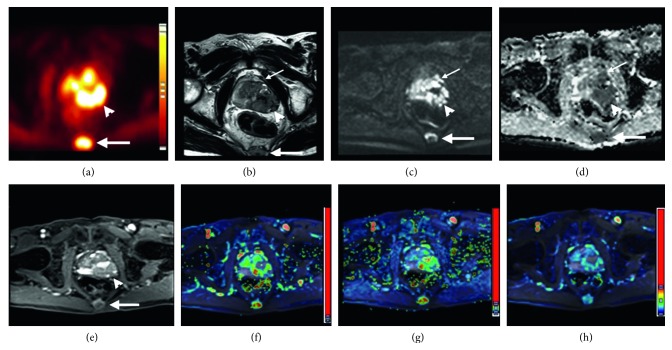
Simultaneous ^11^C-choline PET/MRI images of the prostate region of a 74-year-old man with prostate cancer. (a) PET, (b) T2WI, (c) DWI, b=1000, (d) ADC, (e) DCE, (f) Ktrans, (g) Kep, and (h) iAUC maps derived from DCE showing tumor invasion of the left levator ani muscle (small arrows) and left gross extracapsular extension (arrowheads) by the primary tumor and bone metastasis at the sacrum (large arrows). Liver metastasis is also depicted on PET/MRI (not shown), and the clinical stage is T4N0M1c. Histological examination of the patient's biopsy specimens revealed a Gleason score of 8 and a prostate-specific antigen level of 591.9 ng/ml before PET/MRI.

The simultaneous whole-body PET/MRI acquisition was followed by pelvic PET/MRI scans (Figure 2) involving a pelvic PET scan in one bed position (emission period, 15 min). The MRI pulse sequences included a sagittal T2-weighted TSE sequence (4,000 ms TR/91 ms TE, 4 mm slice thickness, 320 × 224 matrix, and 200 × 200 mm^2^ FOV), coronal T2-weighted TSE sequence (4,000 ms TR/80 ms TE, 4 mm slice thickness, 0.4 mm intersection gap, 256 × 179 matrix, and 180 × 177 mm^2^ FOV), and transverse T2-weighted TSE sequence (3,600 ms TR/80 ms TE, 4 mm slice thickness, 0.4 mm intersection gap, 256 × 179 matrix, and 180 × 177 mm^2^ FOV), and acquisitions were performed in the pelvic region. Axial diffusion-weighted imaging (DWI) was performed using a single-shot spin-echo echo-planar imaging technique under free-breathing conditions (5,000 ms TR/65 ms TE, *b* values of 50 and 1000 s/mm^2^, 4 mm slice thickness, 106 × 106 matrix, 260 × 205 mm^2^ FOV, and NEX 6). Axial DCE-MRI was performed using a 3D T1-weighted spoiled gradient-echo sequence (3.91 ms TR/1.6 ms TE, 4 mm slice thickness, 128 × 128 matrix, 256 × 200 mm^2^ FOV, and 13°FA). A total of 72 volumes were acquired with a temporal resolution of 4.16 seconds and acquisition time of 5 minutes. After four acquisitions of dynamic baseline scanning, a standard dose (0.1 mmol/kg body weight) of gadopentetate dimeglumine (Gd-DTPA; magnevist; Bayer-Schering, Burgess Hill, UK) was administered by a power injector through a cannula placed in the antecubital vein at a rate of 3 mL/s and immediately followed by a saline flush.

Attenuation correction of the PET data was performed using a four-tissue (air, lung, fat, and soft tissue) segmented attenuation map acquired with a two-point Dixon MRI sequence. Images were reconstructed using a high-definition PET (HD-PET) iterative algorithm (three iterations; 21 subsets) with a 5.4 mm post-reconstruction Gaussian filter and an image matrix of 344 × 344.

### 2.3. PET Imaging Parameter Analysis

The PMOD 3.3 software package (PMOD Technologies Ltd., Zurich, Switzerland) was used for tumor segmentation. A volume of interest was manually drawn around the PCa lesion using an SUV cutoff of 2.5 in accordance with previous studies [13, 14]. PET imaging features were calculated in an SUV analysis. The maximum SUV (SUV_max_), mean SUV (SUV_mean_), metabolic tumor volume (MTV), and uptake volume product (UVP) were derived according to the following equations: SUV = (tissue radioactivity/tissue weight (g))/(total radioactivity (Bq)/body weight (g)) and UVP = SUV_mean_ × MTV. Computations for imaging features were performed using the CGITA (Chang Gung Image Texture Analysis) toolbox implemented using MATLAB 2012a (MathWorks, Inc., Natick, MA, USA) [15].

### 2.4. MR Imaging Parameter Analysis

**Figure 3:**
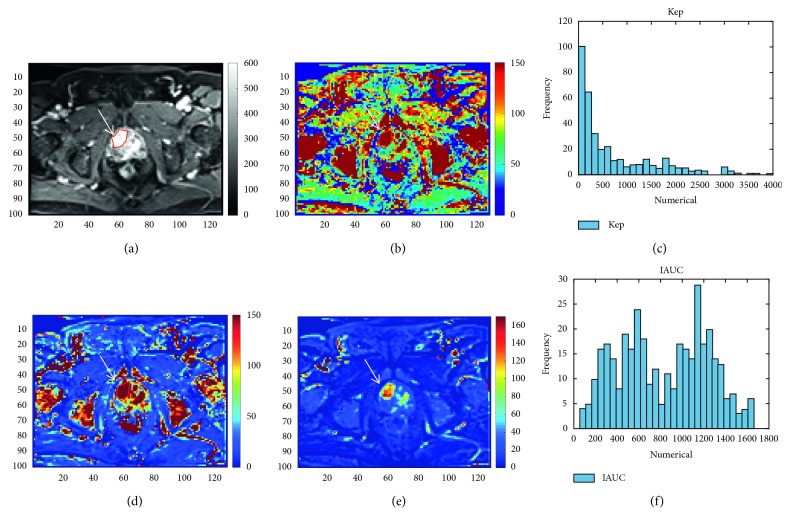
A DCE image and its derived parameter maps at the prostate region of a 76-year-old man who underwent simultaneous ^11^C-choline PET/MRI and had a prostate-specific antigen level of 76.6 ng/ml. A region of interest (ROI) was drawn in the cancer localized at the right lobe of the prostate gland (arrows) to obtain ROI histograms on DCE parameter maps. The Ktrans_max_, Ktrans_kur_, Kep_max_, Kep_kur_, iAUC_max_, and iAUC_kur_ values for prostate cancers in this patient were 3993.0/min, 3.34, 4000.0/min, 4.46, 1716.0, and 1.66, respectively. (a) DCE. (b) Ktrans. (c) Kep histogram. (d) Kep. (e) iAUC. (f) iAUC histogram.

Postprocessing for DWI and DCE-MRI was performed using the software integral to the MRI unit (Siemens Syngo Via and Tissue 4D; software version, VA20B). The ADC was calculated from the diffusion-weighted images. The Toft model [16] was used for pharmacokinetic analysis to derive the following parameters from the DCE-MRI data: the volume transfer rate constant (Ktrans), reflux rate constant (Kep), and initial area under curve (iAUC) in 60 seconds. The ADC- and the DCE-MRI-derived parameter maps were reconstructed on a pixel-by-pixel basis. On axial DCE images, an uroradiologist with over 20 years of experience manually drew the largest region of interest (ROI) within each primary tumor on each image (Figure 3). ROIs on axial ADC images were obtained using a similar approach with the aid of diffusion-weighted images. The histogram data of the derived parameters from the ROIs—including maximal value and kurtosis of Ktrans (Ktrans_max_ and Ktrans_kur_), Kep (Kep_max_ and Kep_kur_), and iAUC (iAUC_max_ and iAUC_kur_)—and the minimal value, mean value, and kurtosis of ADC (ADC_min_, ADC_mean_, and ADC_kur_) were exported for statistical analysis using a homemade software written in MATLAB (R2015b; MathWorks, Inc., Natick, MA, USA).

### 2.5. Statistical Analysis

Descriptive statistics were calculated to summarize the data, using the median (range) for continuous variables and count (percentage of total) for categorical variables. Intergroup comparisons of continuous variables were based on the Mann–Whitney U-test. Correlations among study variables were investigated using the Spearman's correlation method. PFS was defined as the interval between diagnosis and disease progression. Univariate and stepwise multivariate Cox regression analyses based on the forward Wald method, with thresholds of 0.05 and 0.1, respectively, for entering and removing variables, were performed to identify predictors significantly associated with PFS. Two-sided *p* values less than 0.05 were considered statistically significant.

## 3. Results

### 3.1. Patient Clinical Characteristics

The clinical characteristics and PET/MR imaging parameters are listed in Table 1. More than half of the patients were aged ≥70 years. PSA values of 20–50 ng/ml and Gleason scores of 7 were the most common. Clinical T4 tumors were present in more than half of the patients. The majority of patients had clinical stage IV disease, and hypertension was the most common comorbidity.

### 3.2. Correlations among PET/MR Parameters, PSA Levels, and Gleason Scores

Table 2 shows the correlations between PET and MRI parameters. Among the PET imaging parameters, MTV and UVP more frequently showed significant correlations with DCE and ADC parameters. MTV was significantly correlated with Ktrans_max_, Kep_max_, Kep_kur_, iAUC_max_, and iAUC_kur_ (*σ* = 0.39–0.51, all *p* < 0.05), whereas UVP was significantly correlated with Ktrans_max_, iAUC_max_, and iAUC_kur_ (*σ* = 0.37–0.51, all *p* < 0.05). SUV_max_ showed moderate and significant correlation with iAUC_max_. Other SUV and DCE parameters showed no significant correlations. Both MTV and UVP were negatively correlated with ADC_min_ and positively correlated with ADC_kur_. Other SUV and ADC parameters showed no significant correlations. There were no significant correlations between DCE and ADC parameters, except between Ktrans_max_ and ADC_kur_ (*σ*=0.37, *p* < 0.05).

PSA levels were positively correlated with PET imaging parameters, including SUV_max_, SUV_mean_, UVP, and MTV, and negatively correlated with ADC_min_ and ADC_mean_ of MRI. Gleason scores showed positive and significant correlations with both Kep_kur_ and iAUC_kur_.

### 3.3. PET/MR Imaging Parameters Stratified by Clinical Characteristics

The relevant intergroup comparisons of imaging parameters are shown in supplementary Tables S1–S3. All PET parameters but none of the MRI parameters were associated with PSA ≥20 ng/ml (all *p* < 0.05). Three MRI parameters (Kep_kur_, iAUC_kur_, and ADC_mean_) were associated with Gleason scores of 8–10 (all *p* < 0.05). Multiple PET/MRI parameters were associated with the disease stage T3-4, including MTV and UVP from PET, Ktrans_max_, and Kep_max_ from DCE-MRI and ADC_min_ and ADC_kur_ from ADC assessments in MRI (all *p* < 0.05). In contrast, none of the PET/MRI parameters showed significant differences between patients without and with regional lymph node metastasis. Similarly, many PET/MRI parameters (i.e., MTV, UVP, iAUC_kur_, and ADC_min_) showed associations with both distant metastasis and stage IV disease (all *p* < 0.05). SUV_max_ was also associated with distant metastasis, and ADC_mean_, with comorbidities (both *p* < 0.05).

### 3.4. Patients' Disease Progression Predictions by Imaging Parameters

Of the 31 patients, 4 were not treated at our hospital and 27 had undergone radical prostatectomy (*n*=6), radiation therapy (*n*=2), hormone therapy (*n*=11), or radiation plus hormone therapies (*n*=8). The 31 patients had a mean follow-up duration of 25.5 months (range: 7.7–39.0 months). Ten patients (38.5%) showed disease progression at a mean follow-up duration of 16.5 months after diagnosis. Twenty-eight patients (90.3%) were alive at the last visit, and three patients (9.7%) died of prostate cancer at 7.7, 12.0, and 14.0 months after diagnosis.

Univariate Cox regression analysis of solitary and hybrid imaging biomarkers showed that an increase in Kep_max_, Kep_kur_, MTV, UVP, MTV/ADC_min_, UVP/ADC_min_, Kep_max_/ADC_min_, and Kep_kur_/ADC_min_ was significantly associated with a shorter PFS. Stepwise multivariate analysis showed that UVP/ADC_min_ (HR = 1.010, 95% CI: 1.001–1.020, *p*=0.031) and Kep_kur_/ADC_min_ (HR = 1.087, 95% CI: 1.021–1.157, *p*=0.009) were independent predictors of PFS (Table 3).

## 4. Discussion

Unlike biospecimen-based markers, imaging biomarkers undergo technical, biological, and clinical validation along with assessments of cost-effectiveness before they are routinely used in clinical settings. Although PET/MR offers robust data due to the high soft tissue contrast and the addition of multiparametric MRI improves its ability to evaluate prostate cancer in T-staging, the improvements offered by this approach in the detection of nodal disease or bone involvement appear uncertain [17, 18]. Metabolic volumetric PET parameters such as MTV and UVP are considered better than SUV because they provide more accurate PCa characterization [13]. Increased MTV and UVP values reflect the volume of viable tumor cells and high tissue metabolism. In the current study, we performed a comprehensive analysis of the correlations among imaging biomarkers as well as between imaging biomarkers and clinical parameters. The findings showed that MTV and UVP are significantly correlated with many DCE-MRI parameters, which provide tumor blood microcirculation-related information.

Ktrans has been used to quantitatively assess microvascular permeability; Kep reflects the rate of contrast agent transfer from the extravascular extracellular space back to the blood [16]; and iAUC represents the general tumor blood flow, overall perfusion, and tumor interstitial space index. These parameters are correlated with angiogenesis [19, 20]. The correlations among these parameters are not surprising since primary tumors with higher T stages such as ≥T3 have higher MTV, UVP, Ktrans_max_, and Kep_max_ values (Supplementary Tables S1 and S2). Furthermore, MTV and UVP values are also significantly correlated with ADC_min_. ADC values reflect the degree of water restriction, and the increased cellularity of PCa increases water restriction and reduces ADC values. Similarly, patients with distant metastasis also have higher MTV and UVP values but lower ADC_min_ values (Supplementary Table S3). Thus, higher MTV and UVP values may reflect the tumor characteristics of high-risk PCa, not only the higher metabolic activity of larger viable tumors but also the higher angiogenesis and water restriction noted on MRI, and may be useful in categorizing PCa patients by indicating a high likelihood of a higher primary T stage and distant metastasis.

Several promising radiotracers—some of them targeting choline and prostate-specific membrane antigen (PSMA)—are currently being investigated for PET imaging of PCa [21]. An increased cellular membrane synthesis represents a biological substrate for PCa imaging. Specifically, choline enters the cell via the choline transporter, being further phosphorylated to phosphatidylcholine by choline kinase. Both of these molecules are upregulated in tumor cells, ultimately resulting in an enhanced choline uptake [22]. ^11^C-Choline PET imaging parameters, including SUV_max_, SUV_mean_, MTV, and UVP, are significantly correlated with the PSA level, but none of them are correlated with the Gleason score. This may be because several benign entities, especially benign prostate hyperplasia, will also show increased ^11^C-choline uptake [23]. In our data, Kep_kur_ and iAUC_kur_ were correlated with the Gleason score. Kurtosis, as a parameter of first-order histogram analysis, reflects the outliers of a probability distribution in the tail extremity and is considered to be related to tumor heterogeneity [24–26]. Prostate cancers typically have high Ktrans, Kep, and iAUC values on DCE-MRI, and both Ktrans and Kep affect iAUC since the iAUC reflects the overall blood volume of PCa in the initial 60 seconds [27]. The positive correlation of Kep_kur_ and iAUC_kur_ on DCE-MRI with the Gleason score implies that PCa with a more heterogeneous distribution of contrast back to the blood and overall blood volume is more likely to have higher Gleason scores. Thus, ^11^C-choline PET/MRI, in combination with DCE-MRI parameters, provides additional information regarding tumor heterogeneity and Gleason scores of prostate cancers.

Consistently with a previous study conducted with ^18^F-choline [28], we failed to identify a significant inverse association between SUV and ADC values. In contrast, our cases showed a significant inverse correlation between ADC_min_ and UVP/MTV. Decreased ADC values indicate increased water restriction as a result of increased cellularity, and increased MTV and UVP values reflect the volume of viable tumor cells and high tissue metabolism. This correlation may be explained by the fact that histologic tumor volume is significantly associated with both ADC_min_ and metabolic volumetric parameters (as shown in a study conducted with ^18^F-choline) [13]. Combinations of PET and MRI parameters have been considered as imaging biomarkers with the prognostic value in many studies. In one study, the MTV/ADC_min_ ratio was found to be an independent predictor of PFS in pancreatic cancer [29]. Another study demonstrated that percentage changes in SUV_max_/ADC_min_ and tumor lesion glycolysis (TLG)/ADC_min_ can predict treatment response to neoadjuvant chemotherapy (NAC) early in the course of breast cancer treatment [30]. Since multiparametric MRI is a routine clinical tool for prostate cancer diagnosis, the possible prognostic predictive value of PET/MR must be considered. In our study, both UVP/ADC_min_ and MTV/ADC_min_ ratio were significant predictors for disease progression in univariate analysis, and UVP/ADC_min_ was a significant predictor for disease progression in multivariate analysis as Kep_kur_/ADC_min_ (Figure S1).

Our findings need to be interpreted within the context of some limitations. First, most high-risk PCa patients did not undergo prostatectomy, and the number of patients with surgical specimens was small. Although Gleason scores from TRUS biopsies are acceptably accurate in predicting malignancy, 25%–30% of cases may show discrepancies [31]. Second, the treatment was not standardized in all patients. Third, the follow-up durations were relatively short (mean follow-up duration, 25.5 months). These limitations present some uncertainties for the correlation between imaging parameters and biospecimen-based markers and the prognostic significance. However, our study also has several strengths. First, it was the ^11^C-choline PET/MR study focusing on the high-risk PCa staging setting. Second, comprehensive imaging analysis, including PET, DWI, and DCE-MRI, was performed. Third, our data suggested that metabolic volumetric PET parameters, including MTV and UVP, are superior to SUV, show significant correlations with ADC and DCE values, and have prognostic value when combined with ADC_min_.

## 5. Conclusion

The metabolic information provided by ^11^C-choline PET imaging in integrated PET/MR scans shows significant correlations with the PSA level. Metabolic volumetric parameters such as MTV and UVP can serve as imaging biomarkers and show a prognostic value and may show better correlations in combination with MR imaging parameters.

## Figures and Tables

**Table 1:** Clinical characteristics and PET/MR imaging parameters for the patients.

Variables	Descriptive statistics
Age (years)	70 (52–84)
** **<60.0	2 (6.5%)
** **60.0–69.9	11 (35.5%)
** **70.0–79.9	17 (54.8%)
** **≥80	1 (3.2%)

PSA (ng/ml)	30.56 (4.5–591.9)
** **<10	2 (6.5%)
** **10.0–20.0	6 (19.4%)
** **20.1–50.0	11 (35.5%)
** **50.1–100.0	3 (9.7%)
** **>100.0	9 (29.0%)

Gleason score	8 (6–10)
** **6	5 (16.1%)
** **7	9 (29.0%)
** **8	8 (25.8%)
** **9	6 (19.4%)
** **10	3 (9.7%)

Primary T	
** **T2	4 (12.9%)
** **T3a	5 (16.1%)
** **T3b	6 (19.4%)
** **T4	16 (51.6%)

Regional lymph node metastasis	
** **No	17 (54.8%)
** **Yes	14 (45.2%)

Distant metastasis	
** **No	15 (48.4%)
** **Yes	16 (51.6%)^*∗*^

Clinical stage	
** **IIB	2 (6.5%)
** **III	7 (22.6%)
** **IV	22 (71.0%)

PET parameters	
** **SUV_max_	6.36 (4.30–19.43)
** **SUV_mean_	3.63 (2.99–8.03)
** **MTV	14.42 (1.14–156.70)
** **UVP	54.97 (4.14–860.33)

MRI DCE parameters	
** **Ktrans_max_ (1/min)	310.00 (110.00–3993.00)
** **Ktrans_kur_	3.38 (1.64–54.62)
** **Kep_max_ (1/min)	229.00 (66.00–4000.00)
** **Kep_kur_	3.16 (1.73–1640.82)
** **iAUC_max_	1140.00 (635.00–3398.00)
** **iAUC_kur_	2.83 (1.64–11.32)

MRI ADC parameters	
** **ADC_min_ (*µ*m^2^/s)	17.00 (1.00–485.00)
** **ADC_mean_ (*µ*m^2^/s)	1002.78 (674.72–1357.42)
** **ADC_kur_ (*µ*m^2^/s)	3.17 (2.40–5.65)

Descriptive statistics are presented as either median (range) or *n* (%). ^*∗*^Five patients showed both nonregional lymph node and bone metastasis. PSA, prostate-specific antigen.

**Table 2:** Correlation matrix of imaging biomarkers and clinical parameters.

		SUV_max_	SUV_mean_	UVP	MTV	Ktrans_max_	Ktrans_kur_	Kep_max_	Kep_kur_	iAUC_max_	iAUC_kur_	ADC_min_	ADC_mean_	ADC_kur_	PSA	GS
SUV_max_	Correlation	1	0.90	0.69	0.59	0.35	0.15	0.18	−0.04	0.40	0.25	−0.04	−0.19	0.10	0.46	−0.31
	*p* value		<0.01^*∗*^	<0.01^*∗*^	<0.01^*∗*^	0.06	0.41	0.35	0.85	0.03^*∗*^	0.18	0.82	0.31	0.60	0.01^*∗*^	0.10
SUV_mean_	Correlation		1	0.56	0.42	0.19	0.06	−0.02	−0.19	0.28	0.25	0.09	−0.30	0.14	0.39	−0.33
	*p* value			<0.01^*∗*^	0.02^*∗*^	0.30	0.76	0.92	0.31	0.13	0.17	0.64	0.10	0.47	0.03^*∗*^	0.07
UVP	Correlation			1	0.98	0.37	0.31	0.34	0.30	0.51	0.45	−0.41	−0.31	0.37	0.76	0.16
	*p* value				<0.01^*∗*^	0.04^*∗*^	0.09	0.06	0.11	<0.01^*∗*^	0.01^*∗*^	0.02^*∗*^	0.09	0.04^*∗*^	<0.01^*∗*^	0.40
MTV	Correlation				1	0.39	0.25	0.41	0.40	0.51	0.48	−0.47	−0.28	0.40	0.75	0.25
	*p* value					0.03^*∗*^	0.17	0.02^*∗*^	0.03^*∗*^	<0.01^*∗*^	0.01^*∗*^	0.01^*∗*^	0.12	0.03^*∗*^	<0.01^*∗*^	0.18
Ktrans_max_	Correlation					1	1	0.80	0.55	0.60	0.47	−0.24	0.02	0.37	0.18	0.04
	*p* value						<0.01^*∗*^	<0.01^*∗*^	<0.01^*∗*^	<0.01^*∗*^	0.01^*∗*^	0.20	0.93	0.04^*∗*^	0.34	0.83
Ktrans_kur_	Correlation						1	0.74	0.71	0.34	0.78	0.01	−0.09	0.30	0.10	0.25
	*p* value							<0.01^*∗*^	<0.01^*∗*^	0.07	<0.01^*∗*^	0.95	0.64	0.10	0.58	0.17
Kep_max_	Correlation							1	0.80	0.50	0.47	−0.31	−0.04	0.21	0.15	0.21
	*p* value								0.00^*∗*^	0.00^*∗*^	0.01^*∗*^	0.09	0.84	0.26	0.41	0.25
Kep_kur_	Correlation								1	0.32	0.47	−0.26	−0.11	0.24	0.20	0.41
	*p* value									0.09	0.01^*∗*^	0.15	0.57	0.19	0.29	0.02^*∗*^
iAUC_max_	Correlation									1	0.36	−0.25	−0.01	0.26	0.30	0.11
	*p* value										0.046^*∗*^	0.17	0.95	0.16	0.10	0.57
iAUC_kur_	Correlation										1	−0.08	−0.35	0.30	0.33	0.36
	*p* value											0.68	0.05	0.10	0.07	0.049^*∗*^
ADC_min_	Correlation											1	0.14	−0.08	−0.36	−0.33
	*p* value												0.44	0.66	0.046^*∗*^	0.07
ADC_mean_	Correlation												1	−0.20	−0.38	−0.22
	*p* value													0.28	0.04^*∗*^	0.23
ADC_kur_	Correlation													1	0.23	0.35
	*p* value														0.22	0.06
PSA	Correlation														1	0.17
	*p* value															0.35
GS	Correlation															1
	*p* value															

^*∗*^Statistically significant by Spearman's correlation method. PSA, prostate-specific antigen; GS, Gleason score.

**Table 3:** Univariate and multivariate Cox regression for progression-free survival (PFS).

	Univariate analysis				Multivariate analysis			
		95% CI				95% CI		
	HR	Lower	Upper	*p* value	HR	Lower	Upper	*p* value
MTV	1.02	1.00	1.03	0.014^*∗*^	—	—	—	NS
UVP^†^	1.002	1.000013	1.0004	0.049^*∗*^	—	—	—	NS
Kep_max_^†^	1.00046	1.000013	1.0009	0.044^*∗*^	—	—	—	NS
Kep_kur_	1.02	1.00	1.03	0.019^*∗*^	—	—	—	NS
1/ADC_min_	12.52	0.87	180.41	0.063	—	—	—	NS
UVP/ADC_min_	1.01	1.00	1.02	0.007^*∗*^	1.01	1.00	1.02	0.031^*∗*^
MTV/ADC_min_	1.09	1.03	1.14	0.001^*∗*^	—	—	—	NS
Kep_max_/ADC_min_^†^	1.0035	1.001	1.006	0.002^*∗*^	—	—	—	NS
Kep_kur_/ADC_min_	1.09	1.03	1.15	0.002^*∗*^	1.09	1.02	1.16	0.009^*∗*^

^†^Data are expressed as exact numbers; ^*∗*^ *p* value <0.05; NS, not signiﬁcant.

## Data Availability

The data used to support the findings of this study are included within the article.

## Supplementary Materials

Supplementary Materials Tables S1, S2, and S3 demonstrate the relationship between imaging parameters and clinical risk features in PET, DCE, and ADC, respectively. Figure S1 shows Kaplan–Meier plots of progression-free survival according to the hybrid imaging parameters (a) UVP/ADC_min_ and (b) Kep_kur_/ADC_min_. Comparisons were made with the log-rank test. Youden's index was used to determine the optimal cutoff values based on the area under the receiver operating characteristic curves for the events of interest.

## References

[1] Chiang C. J., Lo W. C., Yang Y. W., You S.-L., Chen C.-J., Lai M.-S. (2016). Incidence and survival of adult cancer patients in Taiwan, 2002-2012. *Journal of the Formosan Medical Association*.

[2] Lindenberg L., Ahlman M., Turkbey B., Mena E., Choyke P. (2016). Evaluation of prostate cancer with PET/MRI. *Journal of Nuclear Medicine*.

[3] Umbehr M. H., Muntener M., Hany T., Sulser T., Bachmann L. M. (2013). The role of 11C-choline and 18F-fluorocholine positron emission tomography (PET) and PET/CT in prostate cancer: a systematic review and meta-analysis. *European Urology*.

[4] Hartenbach M., Hartenbach S., Bechtloff W. (2014). Combined PET/MRI improves diagnostic accuracy in patients with prostate cancer: a prospective diagnostic trial. *Clinical Cancer Research*.

[5] O’Connor J. P., Aboagye E. O., Adams J. E. (2016). Imaging biomarker roadmap for cancer studies. *Nature Reviews Clinical Oncology*.

[6] Rakheja R., Chandarana H., DeMello L. (2013). Correlation between standardized uptake value and apparent diffusion coefficient of neoplastic lesions evaluated with whole-body simultaneous hybrid PET/MRI. *American Journal of Roentgenology*.

[7] Schaarschmidt B. M., Buchbender C., Nensa F. (2015). Correlation of the apparent diffusion coefficient (ADC) with the standardized uptake value (SUV) in lymph node metastases of non-small cell lung cancer (NSCLC) patients using hybrid ^18^F-FDG PET/MRI. *PLoS One*.

[8] Shih I. L., Yen R. F., Chen C. A. (2015). Standardized uptake value and apparent diffusion coefficient of endometrial cancer evaluated with integrated whole-body PET/MR: correlation with pathological prognostic factors. *Journal of Magnetic Resonance Imaging*.

[9] D’Amico A. V., Whittington R., Malkowicz S. B. (1998). Biochemical outcome after radical prostatectomy, external beam radiation therapy, or interstitial radiation therapy for clinically localized prostate cancer. *JAMA*.

[10] Roach M., Hanks G., Thames H. (2006). Defining biochemical failure following radiotherapy with or without hormonal therapy in men with clinically localized prostate cancer: recommendations of the RTOG-ASTRO phoenix consensus conference. *International Journal of Radiation Oncology∗Biology∗Physics*.

[11] Hotte S. J., Saad F. (2010). Current management of castrate-resistant prostate cancer. *Current Oncology*.

[12] Thompson I. M., Valicenti R. K., Albertsen P. (2013). Adjuvant and salvage radiotherapy after prostatectomy: AUA/ASTRO guideline. *Journal of Urology*.

[13] Kim Y. I., Cheon G. J., Paeng J. C. (2015). Usefulness of MRI-assisted metabolic volumetric parameters provided by simultaneous (18)F-fluorocholine PET/MRI for primary prostate cancer characterization. *European Journal of Nuclear Medicine and Molecular Imaging*.

[14] Yoneyama T., Tateishi U., Terauchi T., Inoue T. (2014). Correlation of metabolic tumor volume and ^11^C-choline uptake with the pathology of prostate cancer: evaluation by use of simultaneously recorded MR and PET images. *Japanese Journal of Radiology*.

[15] Fang Y. H., Lin C. Y., Shih M. J. (2014). Development and evaluation of an open-source software package “CGITA” for quantifying tumor heterogeneity with molecular images. *BioMed Research International*.

[16] Tofts P. S., Brix G., Buckley D. L. (1999). Estimating kinetic parameters from dynamic contrast-enhanced t1-weighted MRI of a diffusable tracer: standardized quantities and symbols. *Journal of Magnetic Resonance Imaging*.

[17] Eiber M., Rauscher I., Souvatzoglou M. (2017). Prospective head-to-head comparison of (11)C-choline-PET/MR and (11)C-choline-PET/CT for restaging of biochemical recurrent prostate cancer. *European Journal of Nuclear Medicine and Molecular Imaging*.

[18] Souvatzoglou M., Eiber M., Takei T. (2013). Comparison of integrated whole-body [^11^C] choline PET/MR with PET/CT in patients with prostate cancer. *European Journal of Nuclear Medicine and Molecular Imaging*.

[19] Jackson A., O’Connor J. P., Parker G. J., Jayson G. C. (2007). Imaging tumor vascular heterogeneity and angiogenesis using dynamic contrast-enhanced magnetic resonance imaging. *Clinical Cancer Research*.

[20] Oto A., Yang C., Kayhan A. (2011). Diffusion-weighted and dynamic contrast-enhanced MRI of prostate cancer: correlation of quantitative MR parameters with gleason score and tumor angiogenesis. *American Journal of Roentgenology*.

[21] Jadvar H. (2013). Molecular imaging of prostate cancer with PET. *Journal of Nuclear Medicine*.

[22] Piert M., Park H., Khan A. (2009). Detection of aggressive primary prostate cancer with ^11^C-choline PET/CT using multimodality fusion techniques. *Journal of Nuclear Medicine*.

[23] Souvatzoglou M., Weirich G., Schwarzenboeck S. (2011). The sensitivity of [^11^C] choline PET/CT to localize prostate cancer depends on the tumor configuration. *Clinical Cancer Research*.

[24] Just N. (2014). Improving tumour heterogeneity MRI assessment with histograms. *British Journal of Cancer*.

[25] Chen T., Li Y., Lu S. S. (2017). Quantitative evaluation of diffusion-kurtosis imaging for grading endometrial carcinoma: a comparative study with diffusion-weighted imaging. *Clinical Radiology*.

[26] Westfall P. H. (2014). Kurtosis as peakedness, 1905-2014. R.I.P. *American Statistician*.

[27] Yacoub J. H., Oto A., Miller F. H. (2014). MR imaging of the prostate. *Radiologic Clinics of North America*.

[28] Wetter A., Nensa F., Schenck M. (2014). Combined PET imaging and diffusion-weighted imaging of intermediate and high-risk primary prostate carcinomas with simultaneous [^18^F] choline PET/MRI. *PLoS One*.

[29] Chen B. B., Tien Y. W., Chang M. C. (2016). PET/MRI in pancreatic and periampullary cancer: correlating diffusion-weighted imaging, MR spectroscopy and glucose metabolic activity with clinical stage and prognosis. *European Journal of Nuclear Medicine and Molecular Imaging*.

[30] Wang J., Shih T. T., Yen R. F. (2017). Multiparametric evaluation of treatment response to neoadjuvant chemotherapy in breast cancer using integrated PET/MR. *Clinical Nuclear Medicine*.

[31] Fine S. W., Epstein J. I. (2008). A contemporary study correlating prostate needle biopsy and radical prostatectomy gleason score. *Journal of Urology*.

