# Supplementary material for: Metabolic Volumetric Parameters in 11C-Choline PET/MR Are Superior PET Imaging Biomarkers for Primary High-Risk Prostate Cancer
Source: Contrast Media Mol Imaging. 2018 Nov 5;2018:8945130. doi: 10.1155/2018/8945130 (PMC6247714; doi:10.1155/2018/8945130)
Supplement: Supplementary Materials — Tables S1, S2, and S3 demonstrate the relationship between imaging parameters and clinical risk features in PET, DCE, and ADC, respectively. Figure S1 shows Kaplan–Meier plots of progression-free survival according to the hybrid imaging parameters (a) UVP/ADCmin and (b) Kepkur/ADCmin. Comparisons were made with the log-rank test. Youden's index was used to determine the optimal cutoff values based on the area under the receiver operating characteristic curves for the events of interest. [file 8945130.f1.pdf]

Table S1. PET imaging parameters and clinical risk features

	SUVmax				SUVmean				UVP				MTV			
Groups	Median	min	max	p	Median	min	max	p	Median	min	max	p	Median	min	max	p
PSA ≦ 20 (n = 8)	4.99	4.30	6.67	<0.01*	3.33	2.99	3.63	<0.01*	17.26	4.14	145.23	<0.01*	5.47	1.14	45.43	<0.01*
PSA > 20 (n = 23)	7.13	4.37	19.43		4.01	3.18	8.03		94.53	7.14	860.33		19.35	2.17	156.70	
GS ≦ 7 (n = 14)	6.91	4.30	19.43	0.15	3.91	2.99	8.03	0.16	35.65	7.14	860.33	0.57	9.99	2.17	107.21	0.34
GS 8-10 (n = 17)	5.62	4.53	13.31		3.54	3.12	5.30		67.81	4.14	721.20		18.96	1.14	156.70	
T2 (n = 4)	5.25	4.75	7.16	0.26	3.59	3.31	4.28	0.84	18.29	4.14	39.27	0.03*	4.53	1.14	11.88	0.02*
T3-4 (n = 27)	6.46	4.30	19.43		3.81	2.99	8.03		67.81	7.14	860.33		18.96	2.17	156.70	
N0 (n = 17)	6.36	4.30	19.43	0.44	3.61	2.99	6.67	0.60	39.27	4.14	291.22	0.19	11.88	1.14	54.96	0.13
N1 (n = 14)	6.43	4.88	16.95		3.84	3.12	8.03		100.34	10.53	860.33		26.58	2.96	2.96	
M0 (n = 15)	5.41	4.30	11.88	0.04*	3.53	2.99	5.02	0.07	26.04	4.14	202.59	<0.01*	7.06	1.14	44.11	<0.01*
M1 (n = 16)	6.49	5.08	19.43		3.99	3.20	8.03		124.64	13.67	860.33		27.15	3.54	156.70	
Stage II-III (n = 9)	5.62	4.30	11.88	0.22	3.53	2.99	5.02	0.20	21.12	4.14	97.16	0.01*	6.09	1.14	26.22	0.01*
Stage IV (n = 22)	6.41	4.66	19.43		3.91	3.12	8.03		81.17	10.53	860.33		22.70	2.96	156.70	

*P value < 0.05; PSA, prostate-specific antigen; GS, Gleason score.

Table S2. DCE parameters and clinical risk features

	$Ktrans_{max}$				Kep_{max}				Kep_{kur}				$iAUC_{kur}$			
Groups	Median	min	max	p	Median	min	max	p	Median	min	max	p	Median	min	max	p
PSA ≦ 20 (n = 8)	298.0	159.0	1327.0	0.81	278.5	143.0	4000.0	0.67	6.8	2.2	145.0	0.71	2.8	2.3	3.9	0.91
PSA > 20 (n = 23)	310.0	110.0	3993.0		227.0	66.0	4000.0		3.0	1.7	1640.8		2.8	1.6	11.3	
GS ≦ 7 (n = 14)	312.0	133.0	3993.0	0.80	192.0	66.0	4000.0	0.27	2.6	1.7	58.7	0.03*	2.4	1.6	8.5	0.04*
GS 8-10 (n = 17)	276.0	110.0	1327.0		4000.0	78.0	4000.0		8.0	2.2	1640.8		3.2	2.2	11.3	
T2 (n = 4)	163.0	133.0	205.0	0.04*	126.0	66.0	229.0	0.03*	2.5	1.7	5.6	0.15	2.7	2.3	3.6	0.71
T3-4 (n = 27)	320.0	110.0	3993.0		260.0	78.0	4000.0		3.5	1.9	1640.8		2.8	1.6	11.3	
N0 (n = 17)	265.0	110.0	3993.0	0.63	206.0	66.0	4000.0	0.33	3.0	1.7	1640.8	0.15	2.6	1.9	11.3	0.49
N1 (n = 14)	312.0	141.0	1208.0		330.5	141.0	4000.0		8.3	1.9	145.0		3.1	1.6	8.5	
M0 (n = 15)	225.0	110.0	3993.0	0.57	206.0	66.0	4000.0	0.15	2.8	1.7	58.7	0.15	2.4	1.9	3.6	0.05*
M1 (n = 16)	315.0	141.0	1208.0		330.5	99.0	4000.0		8.3	1.9	1640.8		3.6	1.6	11.3	
Stage II-III (n = 9)	164.0	133.0	3993.0	0.85	206.0	66.0	4000.0	0.72	3.5	1.7	58.7	0.51	2.5	1.9	3.3	0.03*
Stage IV (n = 22)	312.0	110.0	1208.0		256.0	78.0	4000.0		3.1	1.9	1640.8		3.3	1.6	11.3	

*P value < 0.05. $Ktrans_{kur}$ and $iAUC_{max}$ were omitted in Table S2 because of a lack of significant results in all comparisons. PSA, prostate-specific antigen; GS, Gleason score.

Table S3. ADC parameters and clinical risk features

	ADC_{min}				ADC_{mean}				ADC_{kur}			
Groups	Median	min	max	p	Median	min	max	p	Median	min	max	p
PSA ≦ 20 (n = 8)	74.0	5.0	283.0	0.23	1060.7	943.4	1357.4	0.11	3.7	2.4	4.7	0.98
PSA > 20 (n = 23)	17.0	1.0	485.0		980.5	674.7	1247.9		3.1	2.4	5.7	
GS ≦ 7 (n = 14)	67.0	2.0	485.0	0.06	1070.3	910.3	1357.4	0.02*	2.8	2.4	4.7	0.11
GS 8-10 (n = 17)	7.0	1.0	269.0		958.2	674.7	1123.0		3.7	2.4	5.7	
T2 (n = 4)	251.0	145.0	485.0	0.01*	947.7	921.2	1247.9	0.76	2.5	2.4	3.8	0.04*
T3-4 (n = 27)	11.0	1.0	462.0		1036.3	674.7	1357.4		3.6	2.4	5.7	
N0 (n = 17)	28.0	2.0	485.0	0.12	1051.4	674.7	1357.4	0.57	2.8	2.4	4.7	0.22
N1 (n = 14)	8.0	1.0	233.0		992.8	859.4	1131.7		3.7	2.4	5.7	
M0 (n = 15)	145.0	2.0	485.0	0.03*	1002.8	674.7	1357.4	0.38	3.1	2.4	4.7	0.57
M1 (n = 16)	6.0	1.0	137.0		1009.5	874.3	1118.7		3.4	2.4	5.7	
Stage II-III (n = 9)	269.0	9.0	485.0	<0.01*	1117.9	921.2	1357.4	0.04*	2.7	2.4	4.7	0.22
Stage IV (n = 22)	6.5	1.0	233.0		973.9	674.7	1131.7		3.6	2.4	5.7	

*P value < 0.05. PSA, prostate-specific antigen; GS, Gleason score.

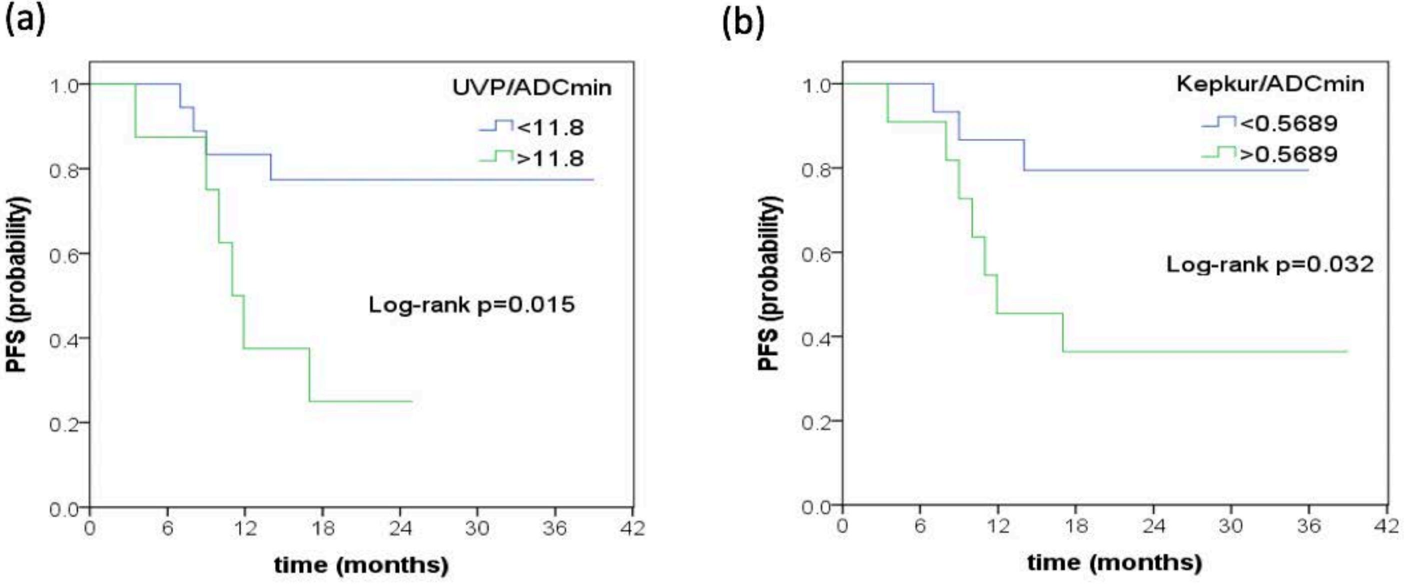
(a)
UVP/ADCmin
<11.8
>11.8
Log-rank p=0.015
PFS (probability)
time (months)
(b)
Kepkur/ADCmin
<0.5689
>0.5689
Log-rank p=0.032
PFS (probability)
time (months)
